# Influence of Context and Setting on the Mental Health and Wellbeing Outcomes of Ayahuasca Drinkers: Results of a Large International Survey

**DOI:** 10.3389/fphar.2021.623979

**Published:** 2021-04-21

**Authors:** Daniel Perkins, Violeta Schubert, Hana Simonová, Luís Fernando Tófoli, José Carlos Bouso, Miroslav Horák, Nicole Leite Galvão-Coelho, Jerome Sarris

**Affiliations:** ^1^ School of Social and Political Science, University of Melbourne, Melbourne, VIC, Australia; ^2^ Independent Researcher, Melbourne, VIC, Australia; ^3^ Interdisciplinary Cooperation for Ayahuasca Research and Outreach (ICARO), School of Medical Sciences, University of Campinas, Campinas, Brazil; ^4^ International Center for Ethnobotanic Education, Research and Service (ICEERS), Barcelona, Spain; ^5^ Medical Anthropology Research Center, Universitat Rovira i Virgili, Tarragona, Spain; ^6^ Department of Neurosciences and Behavior, Ribeirão Preto Medical School, University of São Paulo, Ribeirão Preto, Brazil; ^7^ Faculty of Regional Development and International Studies, Department of Languages and Cultural Studies, Mendel University in Brno, Brno, Czech Republic; ^8^ Postgraduate Program in Psychobiology and Department of Physiology and Behavior, Federal University of Rio Grande do Norte, Natal, Brazil; ^9^ NICM Health Research Institute, Western Sydney University, Westmead, NSW, Australia; ^10^ Professorial Unit, The Melbourne Clinic, Department of Psychiatry, University of Melbourne, Melbourne, VIC, Australia

**Keywords:** ayahuasca, psychedelic therapy, mental health, adverse effects, set and setting

## Abstract

Ayahuasca is a traditional plant decoction containing N,N-dimethyltryptamine (DMT) and various β-carbolines including harmine, harmaline, and tetrahydroharmine, which has been used ceremonially by Amazonian Indigenous groups for healing and spiritual purposes. Use of the brew has now spread far beyond its original context of consumption to North America, Europe, and Australia in neo-shamanic settings as well as Christian syncretic churches. While these groups have established their own rituals and protocols to guide use, it remains unknown the extent to which the use of traditional or non-traditional practices may affect drinkers’ acute experiences, and longer term wellbeing and mental health outcomes. Hence, this study aimed to provide the first detailed assessment of associations between ceremony/ritual characteristics, additional support practices, motivations for drinking, and mental health and wellbeing outcomes. The paper uses data from a large cross-sectional study of ayahuasca drinkers in more than 40 countries who had used ayahuasca in various contexts (*n* = 6,877). It captured detailed information about participant demographics, patterns and history of ayahuasca drinking, the setting of consumption, and ritualistic practices employed. Current mental health status was captured via the Kessler 10 psychological distress scale and the mental health component score of the SF-12 Health Questionnaire, while reported change in prior clinically diagnosed anxiety or depression (*n* = 1276) was evaluated using a (PGIC) Patient Global Impression of Change tool. Various intermediate outcomes were also assessed including perceived change in psychological wellbeing, number of personal self-insights attained, and subjective spiritual experience measured via the spirituality dimension of the Persisting Effects Questionnaire (PEQ) and Short Index of Mystical Orientation. Regression models identified a range of significant associations between set and setting variables, and intermediate and final mental health and wellbeing outcomes. A generalized structural equation model (GSEM) was then used to verify relationships and associations between endogenous, mediating and final outcome variables concurrently. The present study sheds new light on the influence of ceremonial practices, additional supports and motivations on the therapeutic effects of ayahuasca for mental health and wellbeing, and ways in which such factors can be optimized in naturalistic settings and clinical studies.

## Introduction

Ayahuasca is a traditional plant decoction typically made from the *Banisteriopsis caapi (Spruce ex Griseb.) Morton* (ayahuasca) vine and the leaves of *Psychotria viridis Ruiz and Pav* or *Diplopterys cabrerana (Cuatrec.) B. Gates* that contains DMT (N,N-Dimethyltryptamine) along with harmala alkaloids (harmine, harmaline, and tetrahydroharmine), and has the capacity to produce powerful changes in awareness and consciousness. The discovery by scientists and researchers of the therapeutic potentials of ayahuasca is notable as of the early 1990s (McKenna, 2004), and today there is a growing number of studies suggesting potential applications of ayahuasca for the treatment of psychiatric and substance use disorders, as well as broader psychological and spiritual wellbeing benefits for healthy drinkers (Bouso et al., 2012; Barbosa et al., 2016; Uthaug et al., 2018; Dos Santos and Hallak, 2019; Jiménez-Garrido et al., 2020; Mian et al., 2020).

Originally used by Indigenous cultures in the Amazon basin in traditional medicine, from the 1930s the brew was adopted as a religious sacrament by a number of Brazilian syncretic religions, the Santo Daime, União do Vegetal (UDV), and Barquinha (Andritzky, 1989; Halpern et al., 2008). Both the Santo Daime and the UDV have experienced rapid growth since the 1980s and are now present in all major centers in Brazil as well as Europe, North America, and Australia (Shanon, 2002; Tupper, 2009; Trichter, 2010; Lowell and Adams, 2017). A substantial ayahuasca tourism industry has also developed in South American countries where ayahuasca was (and continues to be) traditionally used, with large numbers of international tourists visiting these countries to partake in these ceremonies, primarily for therapeutic and spiritual purposes (Peluso, 2016). Interest in ayahuasca’s purported therapeutic and spiritual effects has also seen the brew widely adopted as part of Indigenous styled neo-shamanic ceremonies taking place in countries across the world (Tupper, 2009). Such adapted ceremonies commonly maintain the basic structure of traditional ceremonies using music and ritual (Trichter, 2010; Gearin, 2015).

Ayahuasca has undoubtedly captured the Western imaginary as a potential therapeutic agent in which there is a symbiosis between the pharmacological properties of the decoction and its traditional ceremonial practices, which makes attention to context, set, and setting particularly relevant to consider. The pharmacology, elemental composition (Guimarães et al., 2020; Kaasik et al., 2020; Orsolini et al., 2020), and how it intertwines with the subjective effects across different contexts continues to be explored, but it is clear that as Giovannetti et al. (2020) note in the case of drug addiction treatment, “ritual elements provide an important space for encouraging the introspection and emotional connection necessary for therapeutic progress.”

The theory of “set and setting” acknowledges a fundamental link between context and positive outcomes, and is well established in the case of psychedelic therapies and interventions (Hartogsohn, 2017; Haijen et al., 2018). Drawing on the initial conceptualization by Leary in the 1960s, “set” refers to the individual and includes “internal, psychological variables such as personality, expectations, suggestibility, preparation, intentions, and mood and psychopathology” while “setting” refers to the “external environment in which the experiences take place, including the physical, interpersonal, and broader social and cultural contexts” (Leary et al., 1963; Breeksema et al., 2020). As Hartogsohn (2016, p.1264) highlights, what is clear is the need to consider “the influence of non-drug/material factors on psychological and medical outcomes effected by material interventions.”

To date, there has been no dataset able to look across different settings to explore the extent to which one or other specific context, set, and setting may be more or less beneficial to the mental health and wellbeing of drinkers. Our study provides a unique opportunity to undertake such analysis using a subset of data from the largest dataset ever collected relating to ayahuasca drinking (*n* = 10,836), which includes individuals from religious, traditional, and other traditional settings in over 50 countries. Here we aim to investigate the influence of set and setting on mental health and wellbeing outcomes of drinkers by considering set at the micro (ceremonial practices and additional supports used) and macro (consumption in a traditional ayahuasca drinking country) or socio-cultural levels, and set in the form of types of motivations reported by drinkers. We explore the degree to which our set and setting measures are associated with mental health and wellbeing directly as well as indirect effects mediated via aspects of the acute ayahuasca experience, and emotional or mental health difficulties reported by drinkers in the weeks or months after consumption.

## Materials and Methods

### Study Design

The cross-sectional data used in this paper were collected between 2017 and 2019 via an online survey that included translations into five languages other than English: Portuguese, Spanish, German, Italian, and Czech. Survey respondents were required to be at least 18°years of age and to have used ayahuasca on at least one occasion. Due to the hidden nature of the ayahuasca drinking population in many countries (where this practice is either prohibited or where its legal status remains unclear), a non-random sampling method was chosen. This enabled the recruitment of a very large number of respondents (*n* = 10,836) that had consumed ayahuasca in traditional, religious and non-traditional settings in more than 50 countries. For the present publication, only participants who had consumed ayahuasca with two or fewer groups were included in the analysis (*n* = 6,877). Survey participation was promoted via websites and email invitations from relevant organizations, ayahuasca retreat centers, and ayahuasca churches, online groups and forums, via Facebook, and flyers at conferences and events. No financial incentives were offered. Data was cross-checked to remove suspected duplicate responses, while data from partially completed surveys was retained. The study was approved by the University of Melbourne Human Research Ethics Committee (HREC number 1545143.3).

### Analysis Framework

**FIGURE 1:**
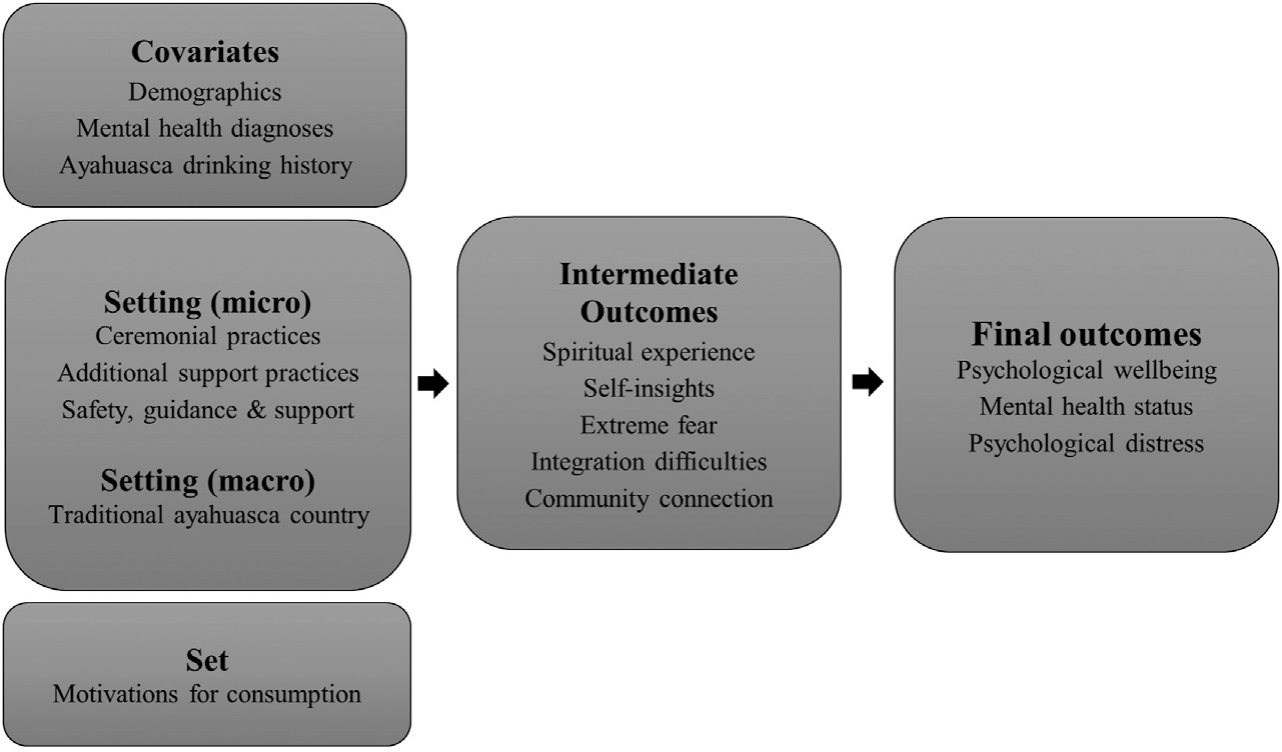
Analysis framework and data items utilized.

As per Figure 1, our approach to this analysis was based on an assumption that set and setting may influence mental health and wellbeing via an effect on what we describe as intermediate outcomes, as well as directly. Although, intermediate outcomes may also have a positive or negative influence on wellbeing in themselves. The inclusion of community connection as an intermediate outcome is in recognition of the influence that set and setting may have, not just on the acute ayahuasca experience and post-ceremony integration, but also in providing opportunities for social engagement and connection. We also include several demographic and ayahuasca drinking history variables, as covariates to attempt to reduce confounding. Other research, for example, has identified the number of times ayahuasca has been consumed as one of the strongest predictors of improved mental health and wellbeing outcomes (Sarris et al., 2021).

### Demographic and Ayahuasca Drinking Variables

Participant demographic information such as age, sex, highest level of education, and country of residence was obtained, as well as number of lifetime mental health diagnoses and detailed information about ayahuasca drinking history and patterns of use. This included the number of times ayahuasca has been consumed, time since last consumption, the country in which most of their drinking had taken place, and the context in which they last drank ayahuasca. The context of consumption provides information about the setting in which ayahuasca was consumed, while the country in which most ayahuasca drinking has taken place will reflect social and cultural contexts that may be an important macro element of setting. We created a variable to identify most drinking occurring within a traditional ayahuasca use country (Peru, Columbia, Ecuador, Bolivia, and Venezuela, but not Brazil), based on the assumption that shaman or other facilitators in these locations would, on average, have access to a more extensive set of skills and knowledge relating to the use of ayahuasca—a difficult to measure component that would not be apparent in ceremonial or other practices used.

To define context respondents were asked whether they had consumed ayahuasca in a ceremony with “one or more traditional shaman” (traditional); with the UDV, Santo Daime or Barquinha churches (ayahuasca church); or with non-traditional, mixed traditional/non-traditional, and no guide or no ceremony/ritual, all of which we included as “Other” contexts. Set information was collected in the form of respondents’ motivations for consuming ayahuasca, selected from a checkbox question with 15 possible choices (see Table 1). In relation to ceremony characteristics respondents were asked to identify all those that were present in their last context of consumption from a check-box list (see Table 1), as well as to rate their experience of six different support, safety and preparation activities on a four-point scale (not at all, a small amount, moderately, very much). Further information was collected about additional support activities that were provided outside of ayahuasca ceremonies (but at the place ayahuasca was consumed), such as religious/spiritual counselling or yoga/tai-chi (see Table 2).

**TABLE 1:** Motivations, ceremony characteristics and additional supports variables.

Motivations	%	Ceremony characteristics	%
Therapeutic motivation (*n* = 6,818)		Traditional (*n* = 6,749)	
To deal with emotional difficulties	37.3	Traditional chants/icaros (live)	37.7
Healing for a mental health condition	21.2	Recommended abstinence of sexual activity prior to ceremonies	34.7
Healing for childhood trauma or abuse	16.1	Blowing of smoke for healing and purification	31.8
Healing for a physical health condition	12.2	Individual blessings	18.5
Healing for adult trauma or abuse	9.1	Whistling	16.7
To assist with grief or loss	8.7	Sucking from affected parts of the body	4.6
Self-knowledge motivation (*n* = 6,818)	%	Non-traditional (*n* = 6,749)	%
For increased spiritual awareness, and understanding or connection	79.4	Smudging (fanning of smoke from burning herbs)	28.7
To gain insight into yourself or parts of your life/self-knowledge	68.8	Other live singing/chanting	22.8
To gain clarity about your life purpose or direction	65.2	Non-traditional instruments (guitar, didgeridoo, harmonica etc)	21.9
To improve personal relationships	35.0	Recitations (prayers, etc.)	18.2
Experiential motivation (*n* = 6,818)	%	Dance	12.9
A general interest in psychedelic medicine or therapy	23.2	Safety and support score (1–4)^a^	Mean score
Curiosity and a desire for adventure	22.7	Empathy, respect and genuineness from those leading/supporting the ceremony (*n* = 6,649)	3.8
For intellectual or creative inspiration	21.8	A sense of emotional and physical safety in the ceremony space (*n* = 6,575)	3.8
To experience the visual effects	12.8	Availability of support during the ceremony if needed (*n* = 6,645)	3.8
Just to have the experience	8.8	A sense of the ceremony as a powerful and sacred ritual (*n* = 6,577)	3.7
		Additional supports	Mean score
		Preparation activities score (1–4)^a^	
		The opportunity to discuss your history, motivations or concerns prior to drinking if desired (*n* = 6,362)	3.1
		Guidance prior to drinking about dealing with potential difficult (*n* = 6,391)	3.1

^a^ responses: 1, not at all; 2, a small amount; 3, moderately; 4, very much.

**TABLE 2:** Motivations, ceremonial, and context variables by reported context of use and region of drinking.

Context of consumption				
	**Ayahuasca church**	**Traditional**	**Other contexts**	**Statistical test**
Motivations				
Therapeutic (0–6)	0.5	1.7	1.7	(F (2, 6,750) = 732.3, *p* < 0.001
Self-knowledge (0–4)	2.3	2.7	2.7	(F (2, 6,759) = 62.6, *p* < 0.001
Experiential (0–5)	0.4	1.4	1.5	(F (2, 6,759) = 714.5, *p* < 0.001
Ceremony characteristics				
Traditional (0–6)	0.2	3.5	2.3	(F (2, 6,698) = 4680.0, *p* < 0.001
Non-traditional (0–5)	0.3	1.7	1.9	(F (2, 6,759) = 714.5, *p* < 0.001
Religious (hymns/sermons)	90.0%	25.3%	26.0%	χ2 (2,N = 6,692) = 2.9e + 03, *p* < 0.001
Safe and supported score (1–4)	3.9	3.8	3.4	(F (2, 6,729) = 314.1, *p* < 0.001
Additional supports				
Preparation activities score (1–4)	3.1	3.3	3.0	(F (2,6,504) = 24.3, *p* < 0.001
Religious/spiritual counseling (y/n)	52.1%	11.2%	9.8%	χ2 (2,N = 6814) = 1.3e + 03, *p* < 0.001
Psychologist/psychotherapist (y/n)	0.8%	7.1%	5.8%	χ2 (2,N = 6814) = 160.8, *p* < 0.001
Yoga/tai-chi (y/n)	1.7%	23.6%	18.3%	χ2 (2,N = 6814) = 654.0, *p* < 0.001
Fasting (y/n)	2.0%	34.9%	25.7%	χ2 (2,N = 6814) = 1.0e + 03, *p* < 0.001
Country of most drinking				
Brazil (n = 3,634)	87.55%	2.2%	10.3%	χ2 (2,N = 6,621) = 3.8e + 03, *p* < 0.001
Other Latin America (*n* = 1,071)	1.8%	60.6%	37.6%	χ2 (2,N = 6,621) = 1.9e + 03, *p* < 0.001
Other countries (*n* = 1,916)	17.3%	27.4%	55.3%	χ2 (2,N = 6,817) = 1.0e + 03, *p* < 0.001
**Region in which most consumption has occurred**				
	**Brazil**	**Other Latin America**	**Other countries**	
Motivations				
Therapeutic (0–6)	0.5	1.7	1.7	(F (2, 6,621) = 817.0, *p* < 0.001
Self-knowledge (0–4)	2.3	2.6	2.7	(F (2, 6,621) = 81.9, *p* < 0.001
Experiential (0–5)	0.4	1.4	1.5	(F (2, 6,621) = 763.2, *p* < 0.001
Ceremony characteristics				
Traditional (0–6)	0.3	3.6	2.3	(F (2,6,594) = 4680.0, *p* < 0.001
Non-traditional (0–5)	0.4	1.4	1.9	(F (2, 6,594) = 714.5, *p* < 0.001
Religious (hymns/sermons)	83.8%	20.7%	35.8%	(F (2,6,555) = 2.0e + 03, *p* < 0.001
Safe and supported score (1–4)	3.9	3.7	3.6	(F (2, 6,538) = 314.1, *p* < 0.001
Additional supports				
Preparation activities (1–4)	3.1	3.2	3.1	(F (2, 6,317) = 6.7, *p* = 0.001)
Religious/spiritual counseling (y/n)	48.5%	13.4%	11.8%	χ2 (2,N = 6,818) = 984.3, *p* < 0.001
Psychologist/psychotherapist (y/n)	1.2%	11.2%	3.9%	χ2 (2,N = 6,814) = 249.5, *p* < 0.001
Yoga/tai-chi (y/n)	2.6%	29.1%	15.7%	χ2 (2,N = 6,814) = 677.5, *p* < 0.001
Fasting (y/n)	2.1%	37.4%	26.3%	χ2 (2,N = 6,818) = 1.1e + 03 *p* < 0.001
Context of consumption				
Ayahuasca church (*n*= 3,529)	87.4%	1.8%	17.3%	χ2 (2,N = 6,621) = 3.8e + 03, *p* < 0.001
Traditional (*n*= 1,255)	2.2%	60.6%	27.4%	χ2 (2,N = 6,621) = 2.0e + 03, *p* < 0.001
Other contexts (n= 1,837)	10.3%	37.6%	55.3%	χ2 (2,N = 6,621) = 1.3e + 03, *p* < 0.001

The number of “self-insights” variable was created based on a list of seven commonly reported personal insights, such as new understanding of childhood events, your physical body function and care, and the purpose and direction of your life (Perkins et al., 2021). This variable displayed a good level of internal consistency (α = 0.80). Such insights are often reported by drinkers to be one of the most profound aspects of their experience (Shanon, 2002). A single item question was used to measure the extent to which extreme fear or panic was experienced during the ayahuasca session, scored from 0 “Not at all” to 10 “Very much.” Individuals were also asked if they have a “close community or network of people” with whom they drink ayahuasca, with responses, Not at all, slightly, moderately, or very much.

### Mental Health, Wellbeing and Spirituality Variables

Several standardized research instruments were used to detect the impact of ayahuasca consumption on mental health and spirituality. The mental component score (MCS) of the SF-12 instrument was used to evaluate current mental health status in accordance with the guidelines for the SF-12 instrument, with scores ranging from 0 to 100 and higher scores signifying better mental health (Maruish, 2012). A second mental health measure, the Kessler-10 (K10) Psychological Distress Scale was also used, with scores ranging from 10 to 50 and a higher number indicating greater psychological distress (Kessler et al., 2003). Perceived growth in psychological wellbeing (PWG) after ayahuasca consumption were measured via the Psychological Wellbeing-Post-Traumatic Changes Questionnaire. This instrument consists of 18 items rated on a five-point Likert scale and measures changes in five areas: self-acceptance, autonomy, purpose in life, relationships, sense of mastery, and personal growth (Joseph et al., 2012). The instrument was chosen due its theoretically grounded approach to measuring change in psychological wellbeing resulting from an extreme event. Although developed in relation to trauma this was seen to be appropriate for ayahuasca due to the ayahuasca experience typically involving an extremely emotionally intense and profound altered state of consciousness, often including highly confronting personal material. Number of “integration difficulties” in the weeks or months after drinking ayahuasca was based on the Patient Health Questionnaire for Depression and Anxiety (PHQ-4) (Kroenke et al., 2009) plus five additional items such as feeling disconnected or alone, and disturbing thoughts, feelings, or sensations.

Respondents were also asked about the presence of lifetime mental health diagnoses and whether they had been experiencing depression or anxiety that had been diagnosed by a health professional at the time of consuming ayahuasca (on any occasion). For those that did report a such a prior disorder a patient Global Impression of Change measure (PGIC; a patient-focused version of the Clinical Global Impressions Scale) (Hurst and Bolton, 2004) was used to identify any perceived effects of ayahuasca drinking on this condition using a six-item scale “much worsened,” “a bit worsened,” “no change,” “a bit improved,” “much improved,” or “completely improved.”

In order to assess subjective spiritual experiences that are commonly reported during psychedelic experiences (Griffiths et al., 2006; Cakic et al., 2010; Griffithsss et al., 2019) the spirituality dimension of the Persisting Effects Questionnaire (PEQ-S) was applied (other dimensions of the PEQ were not used). The PEQ-S uses a single six-point item ranging from “Not at all significant” to “The single most spiritually significant experience of my life.” In addition, the nine-item Short Index of Mystical Orientation (SIMO) was used to explore the intensity of mystical experiences of participants during the acute phase of the ayahuasca experience, with a slight modification replacing the word “god” with “god/spirit/the divine.” This instrument is based on Happold’s seven defining aspects of a mystical experience: ineffability, noesis, transiency, passivity, oneness, timelessness, and true ego (Happold, 1963). Responses to the SIMO were recorded on a scale ranging from 1 (not at all) to 10 (very much) with a total score between 9 and 90 (Francis, 2004).

### Statistical Analysis

Pearson’s χ2 test and one-way ANOVA tests were used to assess differences between categorical and continuous variables. Multivariate models utilized linear regressions for continuous and stereotype logistic regressions for ordinal outcomes, performed using STATA 16. A particular advantage of the stereotype model is that effects of each predictor are not assumed to be identical across the ordinal categories (Fernandez et al., 2019). There was no evidence of multicollinearity for any of the regression models, as assessed by VIF greater than 3 or Pearson’s and Spearman’s correlations greater than 0.7.

To minimize potential bias associated with participants not completing certain questions/sections within the survey we utilized multiple imputation for the imputation of missing data for all variables in multivariate models. We used the mi impute chained command in STATA 16 with imputations undertaken using the regress command for continuous variables, the ologit command for ordinal variables, and logit command for categorical variables with 20 datasets (m = 20) imputed for the analysis.

The GSEM (generalized structural equation modelling) function in STATA 16 was used to identify a final model reflecting a network of causal relationships between motivations, ceremony characteristics, additional supports, wellbeing, and mental health outcomes. GSEM combines generalized linear models and structural equation modeling, to enable simultaneous consideration of direct and indirect effects of interacting factors resulting in a high predictive ability (Lombardi et al., 2017; Mostafa et al., 2020). The model was selected by iteratively adding or removing links to an initial model based on linear model results, theoretical justification, path coefficients significance and minimum information criteria (Tusa et al., 2020).

Exploratory Factor Analyses (EFA) using the principal-component factor method and varimax rotation were undertaken to identify relevant dimensions of respondent motivations, ceremony characteristics, and support activities (see Table 1). Three motivations factors were yielded for use in further analysis (therapeutic, self-knowledge and experiential-see Table 1), along with two ceremony characteristic factors (traditional and non-traditional). The traditional characteristics are consistent with those documented in Amazonian countries (see Beyer, 2009). Two factors relating to preparation safety and support were obtained. All displayed a good level of internal consistency (α **>** 0.7) as assessed by Cronbach’s alpha (Cronbach, 1951). The created motivation and ceremony characteristics variable items were summed to identify the total number of motivations or characteristics selected, while the “safety and support” and “preparation activities” variables used the mean rating (1–4) for each of the included items.

## Results

The sample characteristics by last reported context of consumption are provided in Table 3. Of the full sample 52.1% (*n* = 3,553) had last consumed ayahuasca with an ayahuasca church, 19.6% (*n* = 1,338) in a traditional context, and 28.3% in some “other” context (*n* = 1,926). There was a fairly even split of male and female drinkers in all contexts, with an average age of 40 in ayahuasca church and traditional contexts, and slightly lower in other contexts. A high proportion of drinkers in all contexts had a university education (>60%) in all groups. There were several differences revealed between respondents based on their last context of consumption including the ayahuasca church group being much more likely to reside in Brazil, less likely to have any lifetime mental health diagnosis, and to have drunk ayahuasca a larger number of times (Table 3).

**TABLE 3:** Sample characteristics and ayahuasca drinking variables by reported context of use.

	Ayahuasca church %	Traditional %	Other %	Statistical test
All respondents (n)	52.1 (3,553)	19.6 (1,338)	28.3 (1,926)	
Sex (n)	(3,523)	(1,328)	(1,918)	χ2 (4, N = 6,769) = 6.2, *p* = 0.182
Female	50.7	48.5	50.6	
Male	49.1	51.1	49.3	
Other	0.2	0.5	0.1	
Age (n)	(3,512)	(1,1329)	(1,919)	
Mean age (SD)	40.0 (12.8)	40.3 (11.6)	38.4 (11.5)	(F (2,5,521) = 97.23, *p* < 0.001)
University education (n)	(3,525)	(1,332)	(1,924)	
Yes	63.2	60.4	61.3	χ2 (2, N = 6,781) = 3.80, *p* = 0.150
Region of residence (n)	(3,538)	(1,211)	(1,809)	χ2 (10, N = 6,558) = 3.6e + 03, *p* = 0.000
Brazil	87.0	6.4	19.4	
Other Latin America	0.4	11.1	8.5	
Europe	4.8	41.5	41.0	
North America	6.8	31.9	22.2	
Australia and NZ	0.8	8.0	7.9	
Asia and middle east	0.2	1.2	1.0	
Lifetime mental health diagnosis (*n*)	(2,882)	(1,093)	(1,549)	
Any	29.2	39.0	44.5	χ2 (2, N = 5,524) = 111.3, *p* < 0.001
Number (SD)	0.5 (0.9)	0.8 (1.2)	0.9 (1.3)	(F (2,5,521) = 97.23, *p* < 0.001)
Prior anxiety or depression when drinking ayahuasca	(534)	(274)	(459)	
Yes	19.5	27.1	31.5	χ2 (2, N = 5,214) = 79.0, *p* < 0.001
Number of times drunk (*n*)	(3,281)	(1,331)	(1,913)	χ2 (18, N = 6,525) = 4.3e + 03, *p* < 0.001
1	2.4	18.6	19.8	
2–3	3.2	27.0	30.4	
4–5	1.5	20.4	17.8	
6–10	2.7	17.9	15.4	
11–20	3.3	8.8	8.1	
21–50	8.5	5.2	4.9	
51–100	9.8	1.7	1.7	
101–200	11.7	0.3	0.7	
201–500	28.3	0.2	0.5	
500–5000	28.5	0.1	0.6	
Time since last drank (*n*)	(3,528)	(1,328)	(1,919)	(F (2,6,772) = 4.6, *p* = 0.009)
Number of years (SD)	1.2 (4.7)	1.6 (3.1)	1.2 (2.5)	
Country of most drinking (*n*)	(3,529)	(1,255)	(1,837)	χ2 (10,N = 6,621) = 4.4e + 03, *p* < 0.001
Brazil (*n* = 3,634)	90.1	6.5	20.4	
Other Latin America (*n* = 1,071)	0.5	51.7	21.9	
Europe (*n* = 1,136)	3.6	27.9	35.9	
North America (*n* = 626)	5.4	11.6	15.9	
Australia and NZ (*n* = 117)	0.3	1.4	4.9	
Asia and middle east (*n* = 37)	0.2	1.0	1.0	

Table 2 outlines participants’ motivations for using ayahuasca, as well as ceremony characteristics and additional supports provided by context of consumption and the region in which most of a respondents’ ayahuasca drinking has taken place. Results show that therapeutic and experiential motivation scores were very similar across “traditional” and “other” contexts; and across “other Latin American countries” (excluding Brazil) and “other countries” (outside Latin America). Both groups differed substantially from ayahuasca church. Smaller, but still significant, differences were apparent for the self-knowledge motivation, which was the highest motivation score for each context and region.

A similar pattern was evident for ceremony characteristics where considerable overlap in traditional and non-traditional practices was observed across traditional and other contexts, and “other Latin American” and “other countries,” but again substantial differences were evident compared with “ayahuasca church” context or drinking mostly in Brazil. As expected, the religious characteristic (use of hymns or sermons) was far more commonly reported in the ayahuasca church context and in Brazil, while the safe and supported variable score was also higher in Brazil and the ayahuasca church context. Of the additional supports, preparation activities score was marginally (but significantly) higher in traditional contexts and “other Latin American” countries. While ayahuasca church context/Brazil region was associated with a far greater use of religious/spiritual counseling, and far lower use of psychologist/psychotherapist sessions, yoga/tai-chi, and fasting.

### Multivariate Models

Table 4 reveals significant associations between motivations, ceremony characteristics and additional supports, and the six ayahuasca drinking intermediate outcomes—spiritual experience (SIMO score and PEQ-S), self-insights, experiencing extreme fear, integration difficulties, and a close ayahuasca community). Of the motivations variables, therapeutic motivations were most consistently associated with these outcomes, including positive associations with the number of self-insights attained and subjective spiritual experience rated via the PEQ-S, but also integration difficulties, and extreme fear during an ayahuasca ceremony. Self-knowledge motivation was also positively associated with self-insights and subjective spiritual experience (based on both the PEQ-S and SIMO score), and positively associated with ayahuasca community closeness, but not integration difficulties or experiencing extreme fear. Experiential motivation was negatively associated with spiritual experience (PEQ-S) and ayahuasca community closeness.

**TABLE 4:** Associations between motivations, ceremony characteristics and additional supports and intermediate ayahuasca drinking outcomes.

	Self-insights		SIMO score^e^		PEQ-S^f^		Integration difficulties		Extreme fear		Ayahuasca community^g^	
	B (SE)	P =	B (SE)	P =	B (SE)	P =	B (SE)	P =	B (SE)	P =	B (SE)	P =
Covariates												
Age (decades)	**−0.2 (0.02)**	**0.000**	**−1.88** (**0.18)**	**0.000**	**−0.12** (**0.01)**	**0.000**	**−0.17** (**0.02)**	**0.000**	**−0.22** (**0.03)**	**0.000**	**−0.15** (**0.04)**	**0.001**
Female	−0.1 (0.05)	0.078	0.25 (0.42)	0.554	−0.04 (0.03)	0.194	**0.23** (**0.05)**	**0.000**	0.04 (0.07)	0.575	**0.31** (**0.1)**	**0.002**
University educ	**0.16 (0.05)**	**0.003**	**−1.59** (**0.43)**	**0.000**	**−0.08** (**0.03)**	**0.008**	0 (0.05)	0.967	**0.15** (**0.08)**	**0.041**	−0.01 (0.09)	0.903
Lifetime MH diagnoses^a^	**0.15 (0.03)**	**0.000**	0.41 (0.22)	0.063	0 (0.01)	0.808	**0.18** (**0.03)**	**0.000**	**0.21** (**0.04)**	**0.000**	−0.02 (0.05)	0.714
Num drunk^b^	**0.27 (0.01)**	**0.000**	**1.27** (**0.1)**	**0.000**	**0.09** (**0.01)**	**0.000**	**0.03** (**0.01)**	**0.039**	0 (0.02)	0.877	**0.61** (**0.03)**	**0.000**
Years since drunk^c^	**−0.02 (0.01)**	**0.019**	−0.05 (0.05)	0.359	−0.01 (0)	0.062	−0.01 (0.01)	0.073	0 (0.01)	0.862	**−0.07** (**0.01)**	**0.000**
Motivations												
Therapeutic	**0.16 (0.02)**	**0.000**	0.33 (0.19)	0.081	**0.03** (**0.01)**	**0.022**	**0.15** (**0.02)**	**0.000**	**0.21** (**0.03)**	**0.000**	−0.07 (0.04)	0.077
Self-knowledge	**0.35 (0.02)**	**0.000**	**0.77** (**0.18)**	**0.000**	**0.07** (**0.01)**	**0.000**	−0.01 (0.02)	0.608	0.04 (0.03)	0.196	**0.14** (**0.04)**	**0.001**
Experiential	0 (0.02)	0.949	0.24 (0.19)	0.214	**−0.06** (**0.01)**	**0.000**	−0.01 (0.02)	0.742	0 (0.03)	0.916	**−0.09** (**0.04)**	**0.038**
Ceremony characteristics												
Traditional	0.01 (0.03)	0.685	0.33 (0.2)	0.090	**0.04** (**0.01)**	**0.008**	0.03 (0.03)	0.236	**0.18** (**0.03)**	**0.000**	**−0.16** (**0.04)**	**0.000**
Non-traditional	**0.1 (0.03)**	**0.000**	**0.77** (**0.21)**	**0.000**	0.03 (0.02)	0.065	**0.09** (**0.03)**	**0.000**	**0.1** (**0.04)**	**0.007**	0.05 (0.04)	0.252
Religious	0.09 (0.07)	0.161	**−1.04** (**0.53)**	**0.050**	**−0.08** (**0.04)**	**0.039**	**−0.15** (**0.07)**	**0.028**	**−0.38** (**0.09)**	**0.000**	**0.5** (**0.11)**	**0.000**
Support and safety	**0.1 (0.05)**	**0.050**	**2.42** (**0.42)**	**0.000**	**0.19** (**0.03)**	**0.000**	**−0.13** (**0.05)**	**0.018**	**−0.29** (**0.07)**	**0.000**	**0.73** (**0.11)**	**0.000**
Traditional countries^d^	−0.17 (0.1)	0.074	−0.98 (0.78)	0.209	0.02 (0.05)	0.689	0.15 (0.1)	0.138	**0.7** (**0.14)**	**0.000**	**−1.35** (**0.17)**	**0.000**
Additional supports												
Preparation activities score	**0.22 (0.03)**	**0.000**	**1.51** (**0.25)**	**0.000**	**0.06** (**0.02)**	**0.000**	**−0.14** (**0.03)**	**0.000**	−0.01 (0.04)	0.816	**0.33** (**0.06)**	**0.000**
Religious/spiritual couns	**0.48 (0.06)**	**0.000**	0.07 (0.51)	0.889	0.04 (0.04)	0.262	**−0.23** (**0.06)**	**0.000**	**−0.26** (**0.09)**	**0.004**	**0.4** (**0.12)**	**0.001**
Psychologist/psychotherapist	0.2 (0.15)	0.169	−0.49 (1.16)	0.676	0.02 (0.08)	0.844	0.28 (0.15)	0.059	0.26 (0.2)	0.201	−0.02 (0.24)	0.945
Yoga/tai-chi etc	−0.01 (0.09)	0.888	**1.71** (**0.73)**	**0.020**	**0.11** (**0.05)**	**0.027**	0 (0.1)	0.986	0.13 (0.13)	0.308	−0.02 (0.15)	0.911
Fasting	0.14 (0.08)	0.082	**1.69** (**0.64)**	**0.009**	−0.01 (0.05)	0.813	−0.05 (0.08)	0.545	−0.07 (0.11)	0.566	0.19 (0.13)	0.155
Prob > F (*n*)	*p* < 0.000 (6,877)		*p* < 0.000 (6,877)		*p* < 0.000 (6,877)		*p* < 0.000 (6,877)		*p* < 0.000 (6,877)		*p* < 0.000 (6,877)	

^a^ Number of lifetime mental health diagnoses.

^b^ Lifetime uses of ayahuasca (categories as per Table 1).

^c^ Number of years since last drank ayahuasca.

^d^ Countries include all Amazonian countries where ayahuasca has been used traditionally by indigenous groups except Brazil (Peru, Ecuador, Colombia, Bolivia and Venezuela).

^e^ Strength of the mystical experience.

^f^ Spirituality dimension of the Persisting Effects Questionnaire measuring subjective spiritual experience.

^g^ Drink ayahuasca with a close community.

Of the ceremony characteristics support and safety was significantly associated with all outcomes in a beneficial direction, including a greater number of self-insights, stronger spiritual experience, stronger ayahuasca community, and reduced likelihood of experiencing extreme fear or integration difficulties. Non-traditional ceremony characteristics were associated with increased self-insights and higher SIMO score, but also an increased likelihood of integration difficulties and experiencing extreme fear. Religious characteristics were associated with reduced integration difficulties and experience of extreme fear, and stronger ayahuasca community, but lower spiritual experience (PEQ-S and SIMO score) rating. Traditional practices were associated with a higher spirituality rating on the PEQ-S, but increased likelihood of extreme fear, and lower community rating, similarly drinking ayahuasca in countries where ayahuasca has been traditionally used by indigenous groups was positively associated with experiencing extreme fear and negatively with community closeness.

Among the additional support variables preparation activities score was highly significantly associated with all outcomes (except extreme fear), including a greater number of self-insights, stronger spiritual experience based on the PEQ-S and SIMO score, closer ayahuasca community, as well as reduced integration difficulties. The pattern of associations was similar for “religious or spiritual counselling” excluding spiritual experience (not associated), plus an additional negative association with extreme fear. Having a session with a psychologist/psychotherapist was not associated with any outcomes, while yoga/tai-chi was positively associated with both spirituality measures and fasting with SIMO score only.

Models relating to mental health outcomes (PWG, SF-12 MCS, K10, and PGIC) are presented in Table 5 and again reveal a number of significant associations between these outcomes and drinkers’ motivations, ceremony characteristics, and additional supports provided. Therapeutic motivation was associated with a higher reported growth in PWG, but poorer current mental health (lower SF-12 MCS and higher K10). By contrast self-knowledge motivations were associated with higher reported growth in PWG and better current mental health, measured by the Kessler 10; while experiential motivations were associated with lower reported growth in PWG, as well as poorer current mental health (higher K10 and lower SF-12 MCS), and lower reported improvement in prior anxiety/depression via the PGIC instrument.

**TABLE 5:** Associations between motivations, ceremony characteristics and additional supports and mental health outcomes.

	PWG^e^		SF-12 MCS^f^		Kessler 10^g^		PGIC anx/dep^h^	
	B (SE)	P =	B (SE)	P =	B (SE)	P =	B (SE)	P =
Covariates								
Age (decades)	**−0.67 (0.1)**	**0.000**	**0.81 (0.1)**	**0.000**	**−0.37 (0.05)**	**0.000**	−0.03 (0.02)	0.148
Female	**0.55 (0.24)**	**0.023**	**−1.24 (0.23)**	**0.000**	**0.7 (0.12)**	**0.000**	−0.01 (0.05)	0.825
University educ	**−1.57 (0.25)**	**0.000**	**−1.48 (0.25)**	**0.000**	**0.45 (0.13)**	**0.000**	−0.09 (0.05)	0.077
Lifetime MH diagnoses^a^	0.21 (0.12)	0.087	**−1.05 (0.11)**	**0.000**	**0.73 (0.06)**	**0.000**	0 (0.02)	0.994
Num drunk^b^	**1.2 (0.06)**	**0.000**	**0.53 (0.06)**	**0.000**	**−0.24 (0.03)**	**0.000**	**0.1 (0.01)**	**0.000**
Years since drunk^c^	−0.03 (0.03)	0.253	**−0.07 (0.03)**	**0.019**	0.02 (0.02)	0.208	**−0.02 (0.01)**	**0.011**
Motivations								
Therapeutic	**0.35 (0.11)**	**0.001**	**−0.69 (0.11)**	**0.000**	**0.43 (0.05)**	**0.000**	−0.01 (0.02)	0.687
Self−knowledge	**0.48 (0.1)**	**0.000**	0.07 (0.1)	0.466	**−0.15 (0.05)**	**0.005**	0.03 (0.02)	0.170
Experiential	**−0.65 (0.11)**	**0.000**	**−0.37 (0.1)**	**0.000**	**0.33 (0.06)**	**0.000**	**−0.06 (0.02)**	**0.011**
Ceremony characteristics								
Traditional	−0.22 (0.12)	0.071	−0.19 (0.11)	0.090	**0.16 (0.06)**	**0.005**	**0.05 (0.02)**	**0.031**
Non−traditional	0.12 (0.12)	0.327	−0.02 (0.12)	0.875	−0.05 (0.06)	0.372	0.01 (0.02)	0.706
Religious	−0.12 (0.31)	0.700	0.13 (0.32)	0.679	0.05 (0.16)	0.757	0.01 (0.06)	0.913
Support and safety	**1.91 (0.25)**	**0.000**	**1.5 (0.23)**	**0.000**	**−0.62 (0.12)**	**0.000**	0.07 (0.05)	0.181
Traditional countries^d^	−0.35 (0.48)	0.473	0.58 (0.46)	0.205	**−0.47 (0.22)**	**0.035**	−0.17 (0.09)	0.052
Additional supports								
Preparation activities score	**0.75 (0.14)**	**0.000**	**0.45 (0.14)**	**0.002**	**−0.25 (0.07)**	**0.001**	**0.13 (0.03)**	**0.000**
Religious/spiritual couns	**1.18 (0.29)**	**0.000**	**0.72 (0.28)**	**0.012**	−0.28 (0.15)	0.056	0.06 (0.06)	0.370
Psychologist/psychotherapist	0.59 (0.68)	0.382	**1.39 (0.69)**	**0.044**	**−0.71 (0.35)**	**0.042**	0.06 (0.12)	0.636
Yoga/tai−chi etc	**0.96 (0.44)**	**0.028**	**0.93 (0.43)**	**0.031**	**−0.71 (0.22)**	**0.001**	0.06 (0.08)	0.431
Fasting	**0.97 (0.37)**	**0.008**	0.6 (0.39)	0.129	−0.28 (0.21)	0.183	0.09 (0.07)	0.182
Prob > F (*n*)	*p* < 0.000 (6,877)		*p* < 0.000 (6,877)		*p* < 0.000 (6,877)		*p* < 0.000 (1,278)	

^a^ Number of lifetime mental health diagnoses.

^b^ Lifetime uses of ayahuasca (categories as per Table 1).

^c^ Number of years since last drank ayahuasca.

^d^ Countries include all Amazonian countries where ayahuasca has been used traditionally by indigenous groups except Brazil (Peru, Ecuador, Colombia, Bolivia and Venezuela).

^e^ Perceived growth in psychological wellbeing.

^f^ Current mental health status.

^g^ Psychological distress.

^h^ Reported change in prior clinically diagnosed anxiety or depression.

Of the ceremony characteristics ratings of support and safety were associated with relatively strong positive effects on psychological wellbeing and current mental health (both SF-12 MCS and K10), while traditional ceremony practices were negatively associated with current mental health (higher K10), but positively associated with reported improvement in prior anxiety or depression. Non-traditional and religious ceremony characteristics were not directly associated with any of these outcomes. Drinking ayahuasca in a traditional country was associated with a lower K10 score (reduced psychological distress).

Among the additional supports, the preparation activities score was positively associated with current mental health (higher SF-12 MCS and lower K10), growth in psychological wellbeing, and improvement in prior anxiety or depressive disorders based on the PGIC rating. Religious or spiritual counselling was associated with improved psychological wellbeing and higher SF-12 MCS, while both yoga/tai-chi was positively associated psychological wellbeing growth, as well as better current mental health (higher SF-12 MCS and lower K10), and a session with a psychologist was associated with better mental health based on both the SF-12 MCS and K10. Fasting was positively associated with PWG.

### Proposed Model

**FIGURE 2:**
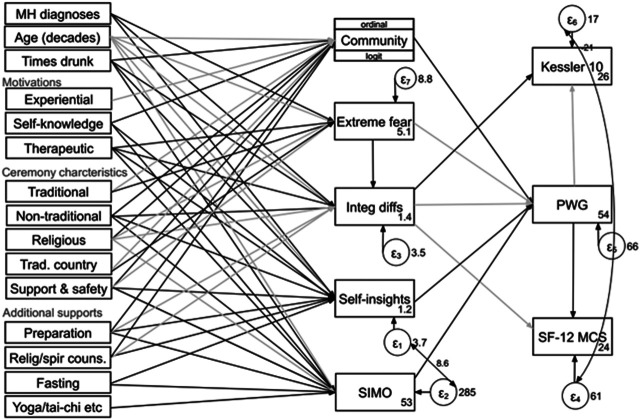
Associations between motivations, ceremony characteristics and additional supports, and intermediate and final mental health outcomes. MH diagnoses, number of lifetime mental health diagnoses; Preparation, preparation activities accompanying ayahuasca use (per Table 1); Relig/spir couns., religious or spiritual counseling; Times drunk, number of times ayahuasca has been drunk; Trad. country, all Amazonian countries where ayahuasca has been used traditionally by indigenous groups except Brazil (Peru, Ecuador, Colombia, Bolivia, and Venezuela). Black color is positively associated, and gray color is negatively associated. All paths shown are significant at *p* < 0.001, other than “Self-knowledge → Community,” “Experiential → Community,” “Relig/spir couns → Integ diffs,” “Therapeutic → SIMO,” “Religious → SIMO,” “Fasting → SIMO,” and “Yoga/tai-chi etc → SIMO,” all *p* < 0.01; and “Fasting → Self-insight,” “Support and safety → Self-insight,” “Non-traditional → Integ diffs” and “Times drunk → Integ diffs” all *p* < 0.05. “Religious → Integ diffs,” and “Non-traditional → Community” was not significant.

Our proposed model of the effects of motivations, ceremony characteristics and additional supports on mental health outcomes is presented in Figure 2. A number of motivation, ceremony and support variables are found to significantly predict the five intermediate outcomes: number of self-insights, strength of the mystical experience (SIMO score), integration difficulties, experiencing extreme fear, and reporting a close ayahuasca community. These five were highly significant predictors of the primary intermediate outcome, reported growth in psychological wellbeing (PWG), which in turn was highly predictive of current mental health status based on both the Kessler 10 and SF-12 MCS. Current mental health status was also negatively predicted directly by integration difficulties (See supporting material Supplementary Table S1 for full model results).

## Discussion

The global spread of the traditional Amazonian psychoactive brew ayahuasca raises questions regarding the role of accompanying cultural and ceremonial practices in ensuring a safe and therapeutic experience for drinkers. Unlike other psychedelic substances currently being investigated for potential clinical application (such as psilocybin), the use of ayahuasca in naturalistic settings almost always takes place as part of a facilitated ceremony or ritual and commonly with a therapeutic intent. However, differing effects of different ceremonial practices have not been previously explored. Our sample of 6,877 ayahuasca drinkers in more than 40 countries who have consumed ayahuasca in religious, traditional and non-traditional settings provides a unique opportunity to investigate such associations.

An interesting higher level finding from our data is that there is considerable fluidity in ceremonial practices across reported contexts of use, with our group of “traditional” characteristics commonly reported in non-traditional contexts, and in countries outside South America. Similarly, our “non-traditional” ceremony practices were relatively common in traditional contexts, although neither group of practices was commonly reported in ayahuasca churches. Such a result is consistent with other research that has highlighted the incorporation of Christian and other spiritual practices and symbols by both traditional indigenous and Mestizo ayahuasca practitioners in South America, as well as the transportation and incorporation of indigenous “shamanic” South American practices and perspectives in ayahuasca healing ceremonies in Western countries (Fotiou, 2012; Labate and Cavnar, 2014; Horák and Verter, 2019; Wolff, 2020).

Multivariate models confirmed associations between ceremonial practices, additional supports, and individual motivations with the six intermediate outcomes considered (self-insights, SIMO score, PEQ-S, integration difficulties, extreme fear, and reporting a close ayahuasca community) and final mental health outcomes (psychological wellbeing growth, SF-12 MCS, K10, and PGIC). Although, in many cases the size of such effects were relatively small individually. Therapeutic motivation was the most consistently associated of the motivation variables, including with a greater number of self-insights, stronger subjective spiritual experience, and higher PWG, but also increased integration difficulties, likelihood of experiencing extreme fear during an ayahuasca ceremony, and poorer current mental health. We believe these latter associations may reflect the nature of this group, with a higher number of therapeutic motivations being a proxy for poorer initial mental/emotional health.

Among ceremony characteristics the support and safety score was the only variable significantly associated with all intermediate and mental health outcomes (and in a therapeutically beneficial direction) other than PGIC, highlighting the importance of a ceremony/ritual process that can provide this experience regardless of the type of other practices used (religious/traditional/non-traditional). As the support and safety score is a rating of participants’ experience of these things during a ceremony, it appears to be identifying passive or active “intangible supports,” defined by Kennedy and Horton (2011) as emotional, structural, moral, spiritual, or other interpersonal forms of encouragement or support. These appear to play a crucial role in optimizing drinkers experience and outcomes. Non-traditional practices were also associated with intermediate benefits in-terms of self-insights, spiritual experience, and ayahuasca community, but also a slight increase in integration difficulties, with no detectable effects on final mental health outcomes. Traditional practices were associated with a higher rated spiritual experience (PEQ-S), but also increased extreme fear, and reduced community connection (likely because people who travel to South America to use ayahuasca usually spend only a limited time in a retreat center), poorer current mental health (SF-12 MCS and K10), but greater PGIC reported improvement. Religious characteristics were not significantly associated with any mental health outcomes.

Of the additional support practices considered, the importance of adequate preparation activities was highlighted via the significant (beneficial) associations between this item and all intermediate and final mental health outcomes (other than extreme fear). A similar but somewhat less consistent benefit was observed for religious or spiritual counselling, while effects of a session with a psychologist or psychotherapist were apparent only for final outcomes, better current mental health (lower K10 and higher SF-12 MCS). The broad apparent benefit of religious or spiritual counselling is an interesting finding and not surprising given the profound and often life changing spiritual content commonly present within ayahuasca experiences. It also raises a question about whether a psychospiritual framework, such as Internal Family Systems therapy, may improve therapeutic outcomes, compared with standard Western psychotherapeutic approaches (Sweezy et al., 2013; Morgan, 2020). This may also apply to the clinical use of related substances such as psilocybin that have a similarly powerful spiritual dimension.

The use of body therapies such as yoga/tai-chi was positively associated with both spirituality measures, greater psychological wellbeing growth, and better current mental health (SF-12 MCS and K10), and in this regard we note experimental research with ayahuasca that has identified significant activation of neural systems involved with interoception (particularly frontal and paralimbic areas) (Riba et al., 2006). It seems feasible that physical therapies such as yoga or tai-chi, which support somatic awareness (Impett et al., 2006) may work synergistically by allowing greater connection to bodily sensations and subtle messages during the acute ayahuasca experience, and possibly post-ceremony during the integration of emotional and psychological material.

A notable aspect of our study is to illuminate the pathways by which ceremonial practices, additional supports and individuals motivations affect the mental health and wellbeing of ayahuasca drinkers, and the key mediating role of aspects of the acute experience (spiritual experience, self-insights, extreme fear) and, integration difficulties in the weeks or months after drinking ayahuasca. These factors are all strong predictors of perceived growth in psychological wellbeing, which in turn was highly predictive of current mental health status. Moreover, unlike previous studies, we are also able to identify and consider separately the sizable wellbeing and mental health benefits associated with the social and community aspects of ayahuasca drinking, on which ceremony practices, additional support, and drinkers motivations also have an influence.

Our study has a number of important strengths, including a large sample size, international cross-cultural sampling frame, and inclusion of ayahuasca drinkers from a range of different contexts of consumption. However, several limitations are also important to note. In particular, the use of a non-random, self-selected sample that risks bias towards drinkers experiencing positive effects, who are motivated to spend time completing a survey, compared with those who had negative or neutral experiences, who may feel lower impetus for involvement. The data collection instrument was entirely self-report, and several items involved retrospective assessments, although this did not apply to the mental health measures SF-12 MCS and K10. The cross-sectional study design also means that we do not have data relating to the mental health status of respondents prior to their consumption of ayahuasca, and there is a possibility that individuals with better mental health were more likely to report improved psychological wellbeing, and hence that better current mental health status may simply be associated with better past mental health. We have included number of lifetime mental health diagnoses as a proxy for mental wellbeing, to adjust for such effects, however this may not be a completely satisfactory substitute. Additionally, we do not have data regarding the composition, purity, and dose (concentration and quantity) of the ayahuasca being consumed, and hence potential influence of these factors. However, we would expect a higher dose to be reflected in higher subjective spiritual experience scores.

It is also important to note that while specific practices may influence the experiences and mental health and wellbeing outcomes of ayahuasca drinkers, there are potentially other aspects of the ceremony and additional supports that are of equal or greater importance. Our inclusion of a variable identifying ayahuasca consumption in countries for which ayahuasca has been traditionally used by indigenous groups was intended to capture ayahuasca use that was more likely to involve shaman/facilitators with a higher level of experience, knowledge and skill; although this is not always the case as some tourist focused centers use shaman with little training, and some people in western countries attend ceremonies hosted by traditional Amazonian shaman “touring” in their country. In addition, our data regarding set was limited to only the motivations of drinkers, while research with other psychedelics such as psilocybin identifies various aspects of an individual’s mental state at the time of ingestion as being predictive of both mystical and adverse experiences during the acute phase as well as longer term mental health outcomes (Russ et al., 2019). It would be of benefit for future research to attempt to incorporate such elements, as well as obtaining greater specificity in areas we have identified as being important, such as support and safety.

**FIGURE 3:**
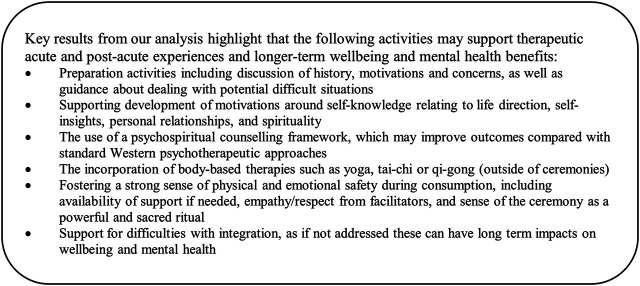
Summary of key findings.

In conclusion, we identify that aspects of setting (ceremonial practices and additional supports) and set (individual motivations) appear to have significant effects on drinkers’ acute experiences, likelihood of experiencing difficulties with integration, and longer term wellbeing and mental health outcomes. Noting the complexity in attempting to quantify and measure the multifactorial inputs relating to the consumption of ayahuasca in ceremonial contexts, we identify limited evidence for the superiority of one specific set of practices. However, tangible and intangible supports that provide a sense of safety and support in the ceremonial context, and preparation activities were found to be particularly important. Figure 3 provides a summary of key findings of potential interest for the therapeutic use of ayahuasca in naturalistic and clinical settings.

## Data Availability

The datasets presented in this article are not readily available because Ethics approval and consent signed by participants was for data access by research team members only. Requests to access the datasets should be directed to d.perkins@unimelb.edu.au.
