# Supplementary material for: Influence of Context and Setting on the Mental Health and Wellbeing Outcomes of Ayahuasca Drinkers: Results of a Large International Survey
Source: Front Pharmacol. 2021 Apr 21;12:623979. doi: 10.3389/fphar.2021.623979 (PMC8097729; doi:10.3389/fphar.2021.623979)
Supplement: Supplementary file 1 [file table1.docx]

**S1. Table.1 Generalised Structural Equation Model (GSEM) of motivations, ceremony characteristics and additional supports, intermediate and final mental health and wellbeing outcomes of drinkers (n=6,402)**

|  | **Coef.** | **Std. Err.** | **z** | **P>z** | **[95% Conf.** | **Interval]** |
| --- | --- | --- | --- | --- | --- | --- |
| **Self-insight (n= 4,892)** |  |  |  |  |  |  |
| Non-traditional | 0.114 | 0.024 | 4.780 | 0.000 | 0.067 | 0.160 |
| Fasting | 0.157 | 0.080 | 1.970 | 0.049 | 0.001 | 0.314 |
| Times drunk | 0.288 | 0.012 | 24.300 | 0.000 | 0.265 | 0.311 |
| Preparation | 0.210 | 0.032 | 6.480 | 0.000 | 0.146 | 0.274 |
| Support & safety | 0.126 | 0.055 | 2.310 | 0.021 | 0.019 | 0.234 |
| Age (decades) | -0.219 | 0.023 | -9.450 | 0.000 | -0.265 | -0.174 |
| Therapeutic | 0.167 | 0.024 | 7.110 | 0.000 | 0.121 | 0.213 |
| Self-knowledge | 0.337 | 0.023 | 14.570 | 0.000 | 0.292 | 0.383 |
| MH diagnoses | 0.123 | 0.026 | 4.830 | 0.000 | 0.073 | 0.174 |
| Relig/spir couns. | 0.480 | 0.066 | 7.280 | 0.000 | 0.350 | 0.609 |
| _cons | 1.246 | 0.202 | 6.160 | 0.000 | 0.850 | 1.643 |
| **SIMO (n= 4,892)** |  |  |  |  |  |  |
| Non-traditional | 0.928 | 0.193 | 4.800 | 0.000 | 0.549 | 1.307 |
| Fasting | 1.782 | 0.643 | 2.770 | 0.006 | 0.521 | 3.043 |
| Times drunk | 1.246 | 0.100 | 12.430 | 0.000 | 1.049 | 1.442 |
| Preparation | 1.519 | 0.267 | 5.700 | 0.000 | 0.997 | 2.041 |
| Support & safety | 2.220 | 0.448 | 4.960 | 0.000 | 1.342 | 3.097 |
| Age (decades) | -2.183 | 0.188 | -11.590 | 0.000 | -2.552 | -1.814 |
| Therapeutic | 0.517 | 0.176 | 2.940 | 0.003 | 0.172 | 0.862 |
| Self-knowledge | 0.849 | 0.185 | 4.580 | 0.000 | 0.486 | 1.212 |
| Religious | -1.387 | 0.530 | -2.620 | 0.009 | -2.425 | -0.348 |
| Yoga/tai-chi etc | 2.099 | 0.711 | 2.950 | 0.003 | 0.704 | 3.493 |
| _cons | 53.322 | 1.628 | 32.760 | 0.000 | 50.132 | 56.512 |
| **Integ diffs (n= 4,583)** |  |  |  |  |  |  |
| Extreme fear | 0.203 | 0.009 | 21.690 | 0.000 | 0.184 | 0.221 |
| Non-traditional | 0.061 | 0.024 | 2.530 | 0.011 | 0.014 | 0.108 |
| Times drunk | 0.030 | 0.013 | 2.290 | 0.022 | 0.004 | 0.055 |
| Preparation | -0.151 | 0.029 | -5.200 | 0.000 | -0.208 | -0.094 |
| Age (decades) | -0.143 | 0.024 | -6.030 | 0.000 | -0.190 | -0.097 |
| Therapeutic | 0.095 | 0.023 | 4.070 | 0.000 | 0.049 | 0.141 |
| MH diagnoses | 0.125 | 0.027 | 4.620 | 0.000 | 0.072 | 0.178 |
| Relig/spir couns. | -0.194 | 0.069 | -2.800 | 0.005 | -0.330 | -0.058 |
| Religious | -0.132 | 0.070 | -1.880 | 0.060 | -0.270 | 0.005 |
| _cons | 1.437 | 0.158 | 9.090 | 0.000 | 1.128 | 1.747 |
| **Community (n= 4,553)** |  |  |  |  |  |  |
| Non-traditional | 0.037 | 0.027 | 1.370 | 0.171 | -0.016 | 0.090 |
| Times drunk | 0.419 | 0.016 | 25.990 | 0.000 | 0.388 | 0.451 |
| Preparation | 0.193 | 0.039 | 4.960 | 0.000 | 0.117 | 0.270 |
| Support & safety | 0.415 | 0.065 | 6.380 | 0.000 | 0.287 | 0.542 |
| Age (decades) | -0.113 | 0.028 | -4.060 | 0.000 | -0.167 | -0.058 |
| Self-knowledge | 0.080 | 0.027 | 2.950 | 0.003 | 0.027 | 0.133 |
| Relig/spir couns. | 0.299 | 0.085 | 3.510 | 0.000 | 0.132 | 0.465 |
| Traditional | -0.105 | 0.025 | -4.260 | 0.000 | -0.153 | -0.056 |
| Religious | 0.280 | 0.074 | 3.780 | 0.000 | 0.135 | 0.425 |
| Experiential | -0.077 | 0.026 | -2.980 | 0.003 | -0.128 | -0.026 |
| Trad. country | -1.022 | 0.100 | -10.260 | 0.000 | -1.217 | -0.827 |
| **PWG (n= 4,491)** |  |  |  |  |  |  |
| Self-insights | 1.452 | 0.069 | 21.150 | 0.000 | 1.318 | 1.587 |
| SIMO | 0.176 | 0.008 | 23.170 | 0.000 | 0.161 | 0.191 |
| Integ diffs | -0.463 | 0.076 | -6.070 | 0.000 | -0.612 | -0.313 |
| Extreme fear | -0.359 | 0.043 | -8.330 | 0.000 | -0.443 | -0.274 |
| Community | 1.799 | 0.112 | 16.060 | 0.000 | 1.580 | 2.019 |
| _cons | 54.469 | 0.553 | 98.410 | 0.000 | 53.384 | 55.553 |
| **SF-12 MCS (n= 4,168)** |  |  |  |  |  |  |
| Integ diffs | -1.366 | 0.055 | -24.660 | 0.000 | -1.474 | -1.257 |
| PWG | 0.362 | 0.012 | 31.090 | 0.000 | 0.339 | 0.384 |
| _cons | 24.343 | 0.904 | 26.920 | 0.000 | 22.570 | 26.116 |
| **Kessler 10 (n= 4,252)** |  |  |  |  |  |  |
| Integ diffs | 0.840 | 0.034 | 24.980 | 0.000 | 0.774 | 0.906 |
| PWG | -0.170 | 0.006 | -27.740 | 0.000 | -0.182 | -0.158 |
| _cons | 25.841 | 0.479 | 53.980 | 0.000 | 24.903 | 26.779 |
| **Extreme fear (n= 5,084)** |  |  |  |  |  |  |
| Support & safety | -0.406 | 0.076 | -5.350 | 0.000 | -0.554 | -0.257 |
| Age (decades) | -0.184 | 0.035 | -5.330 | 0.000 | -0.252 | -0.116 |
| Therapeutic | 0.225 | 0.036 | 6.280 | 0.000 | 0.155 | 0.295 |
| MH diagnoses | 0.194 | 0.041 | 4.730 | 0.000 | 0.114 | 0.275 |
| Traditional | 0.246 | 0.032 | 7.800 | 0.000 | 0.184 | 0.308 |
| Religious | -0.394 | 0.102 | -3.870 | 0.000 | -0.593 | -0.195 |
| Trad. country | 0.694 | 0.144 | 4.820 | 0.000 | 0.411 | 0.976 |
| _cons | 5.085 | 0.318 | 15.970 | 0.000 | 4.461 | 5.709 |
